# Crafting Life Stories in Photocollage: An Online Creative Art-Based Intervention for Older Adults

**DOI:** 10.3390/bs12010001

**Published:** 2021-12-21

**Authors:** Shoshi Keisari, Silvia Piol, Talia Elkarif, Giada Mola, Ines Testoni

**Affiliations:** 1 Department of Philosophy, Sociology, Pedagogy and Applied Psychology (FISPPA), University of Padova, 35131 Padova, Italy; silvia.piol.1@studenti.unipd.it (S.P.); giada.mola@studenti.unipd.it (G.M.); ines.testoni@unipd.it (I.T.); 2 School of Creative Arts Therapies, University of Haifa, Haifa 3498838, Israel; tali.elkarif@gmail.com; 3 Emili Sagol Creative Arts Therapies Research Center, University of Haifa, Haifa 3498838, Israel

**Keywords:** tele-psychotherapy, tele-arts therapy, creative arts therapies, older adults, photocollage, narrative gerontology

## Abstract

Creative arts therapies (CAT) provide a safe and creative environment for older adults to process life experiences and maintain personal growth while aging. There is a growing need to make creative arts therapies more accessible to the aging population, as many have limited access to these services. This need has been catalyzed by the advent of the COVID-19 pandemic. Tele-CAT offers a possible solution. This study explored the experiences of older adults who participated in an online creative process of digital photocollage based on CAT. Twenty-four Italian and Israeli community-dwelling older adults aged 78 to 92 participated in this research through Zoom teleconferencing software. Transcriptions of the sessions and the art produced through the photocollage were qualitatively analyzed through Thematic Analysis. The findings show that the projective stimuli of digital photographs supported older adults’ narratives and engaged them in a more embodied emotional experience. Participant experiences involved artistic enjoyment within a positive and safe interaction with therapists. The creation of digital photocollages allowed the participants to process their life experiences and create an integrative view of their life, a vital developmental task in late life. These results point to the advantages and challenges of tele-CAT for older adults.

## 1. Introduction

Creative arts therapies (CAT) is an umbrella term for healthcare professions employing the creative and expressive process of art making to foster the psycho-social well-being of individuals [1]. CAT include a whole spectrum of disciplines, such as art therapy, drama therapy, psychodrama, dance/movement therapy, music therapy and poetry therapy, all of which foster the creative processing of emotions and experiences. The therapeutic processes are led by certified arts therapists and are grounded in the triangular relationship between the therapist, the client, and the art process and product [2]. As in other psychotherapies, establishing a safe environment and therapeutic alliance are central factors in the therapeutic relationship in CAT [3]. The aim of the current study was to explore the experiences of older adults who participated in an online therapeutic creative process of digital photocollage as a tele-CAT intervention during a period of social distancing. More specifically, we aimed to better understand how the nature of CAT can be integrated into an online format with older adults.

There are several change factors in CAT which distinguish it from verbal psychotherapy [3]. During the creative process of art making, the physical senses are engaged through relationships with paint, clay, images, movements and sounds. This embodied sensual experience fosters the connection and the working through of emotions rooted in bodily experience [4]. This emotional awareness can be further communicated in verbal and non-verbal ways in the therapeutic encounter [2]. The sensory experience also involves pleasure and relief that makes the exploration of difficult content tolerable [4].

In the therapeutic process of CAT, symbols and metaphors are created and explored [5]. This enables individuals to succinctly craft their inner experiences and express their invisible dimensions [6]. The use of colors, shapes and bodily movements also facilitates *concretization,* the transformation of abstract internal psychological content into an externalized tangible artistic presentation [7]. Artistic expression, through its symbolic concrete language, helps individuals bring the subconscious into conscious awareness and to communicate it with the self and others [8]. This is also connected to the concept of *aesthetic distancing*, one of the main change factors of CAT, which refers to an individual’s ability to simultaneously be emotionally *close* enough to the artistic experience yet *distant* enough to cognitively reflect upon it [9]. Aesthetic distancing provides a safe space for observing and exploring the emotional experience during the creative process.

Art making is also based on spontaneity, which brings individuals closer to their emotions, thoughts, and imagination [10]. Spontaneity includes vitality, intuition, and the ability to accept each moment as it comes and to respond dynamically to it [11]. Spontaneity induces creativity, which strengthens people’s ability to look at a familiar experience from new and different perspectives [10,12]. The literature points to an association between creativity and well-being indicators such as adjustment, optimal functioning, and the ability to respond adaptively to stressful situations and changes in life [13].

CAT are also action-oriented interventions that promote active involvement in the creative process, since the participants are actively and physically involved in the process of creating something visible [14,15]. This process positions the participants in the active positions of “artists” and “creators”, rather than in the passive roles of “patients” or “clients”. In this way, CAT enable individuals to cope better with the stigma attached to mental health services, especially in older populations [16,17].

### 1.1. Creative Arts Therapies with Older Adults

There is a growing body of literature concerning the use of creative arts therapy interventions with older adults, and its effectiveness in improving various aspects of mental health [17,18,19,20,21]. The Dunphy and colleagues (2019) review showed that CAT can support older adults in dealing with depressive symptoms [20]. It pointed to various change factors in the creative therapeutic process that lead to its effectiveness in treating depression that include enhancing emotion processing, increasing social bonding, fostering creative expression and aesthetic pleasure. In addition, CAT for older adults affected physiological and cognitive measurements (e.g., by acting on neurochemicals such as endorphins, and stimulating memories).

Studies show that visual images provide a non-verbal means of expression in the therapeutic process with older adults. The ability to express oneself through these media is also important in cases of decreased cognitive functioning [22]. A review study underscored the efficiency of visual arts therapy in promoting psychological and cognitive well-being among older adults who suffer from decreased cognitive functioning [23]. Studies have shown that using visual images such as photography [24,25], collages [26], drawing, and video recording [27,28] can be effective when processing the memories and life stories of older adults. Studies have also shown that creative arts therapies support older adults in processing their life stories and unfinished business from the past [24,29,30].

Overall, there is a growing need to make mental health services accessible for older adults, since many have limited access to these services generally [31,32,33,34,35]. The COVID-19 pandemic with its social restrictions catalyzed this need and underscored the importance of supporting the older population in their homes [36]. Tele-psychotherapy increases the accessibility of mental health services. Thus, it is crucial to develop tele-CAT for the aging population that will make arts-based interventions more accessible, and at the same time maintain the creative nature of the therapeutic process in the online setting.

### 1.2. Tele Psychotherapies and Tele-CAT for Older Adults

Tele-psychotherapy is a form of therapy that is conducted through a digital device, such as a smartphone, tablet or computer, by means of a videoconferencing application (such as Zoom, Skype, Meet) or via the telephone. Tele-psychotherapy can overcome some of the barriers that prevent older adults and the general public from turning to psychotherapy, such as physical limitations, lack of transportation, financial difficulties, and social stigma [37,38,39]. This is extremely important in the case of older adults whose mobility difficulties can lead to social isolation, which is associated with a decline in mental health [40,41]. Sometimes, patients feel more comfortable when engaging in therapy from their homes [42]. Nevertheless, therapists have some concerns about tele-psychotherapy [43,44], which mainly relate to the lack of user-friendliness with the software, difficulties establishing and maintaining the client-therapist therapeutic alliance, lack of confidentiality, and doubts as to its efficacy.

Despite these hesitations, studies show that tele-psychotherapy is effective in treating older adults. A randomized controlled trial by Shapira and colleagues (2021) showed the potential of a brief group online intervention to reduce loneliness and depressive symptoms in older adults during the pandemic [33]. In a recent study by Choi et al. (2020), tele-psychotherapy was found to improve social interactions among homebound older adults. The participants reported that they experienced the therapy positively, appreciated the opportunity to engage with a professional online, expressed feelings of productivity and hope for the future, and felt a sense of achievement from the tele-psychotherapy [38]. However, despite the development of tele-psychotherapy for the aging population, it is difficult to find studies that have examined the outcomes of tele-CAT for older adults.

There is a growing body of studies on tele-CAT and the use of digital resources in the creative process [45,46,47]. A recent review of the literature described the advantages and disadvantages of art therapy within the digital space [47]. The findings show that similar to tele-psychotherapy, tele-CAT bridge geographical distances, and cope better with mobility disabilities and the stigma related to mental health services. Visual images can be shared online, and the artworks can be endlessly modified. The use of technology in therapy has been found to be helpful in reducing client resistance to therapy and/or art making. The client’s home environment involvement in the therapy provides an opportunity to establish deeper trust in the therapeutic relationship. This literature review also points to several concerns, such as the lack of a sensual and tactile quality in the digital art or the limited space and techniques for art making. Other concerns related to ethics, resistance to digital arts media, and technological limitations. While this review noted the increased use of digital technology by art therapists for both online delivery and digital art making, there is still scant literature on the ability to deliver tele-CAT for older adults [48]. The present study describes an online creative intervention for community-dwelling older adults based on the integration of photocollage with narrative elements of Dignity Therapy (DT).

### 1.3. An Online Creative Process with Photocollages Based on Dignity Therapy

A collage is a form of art therapy in which participants select, arrange, and attach various materials to a blank surface [49,50,51]. Transforming a collection of photographs into new form can be freeing, allowing new perspectives to be discovered [52]. Creating collages is effective in processing memories and life experiences in the older population [26], which is an important developmental task in old age [53,54]. As various life experiences are visualized together and assembled in the same art product, crafting the photocollage can help provide a more integrative view of the self. As Petit (2005) noted with respect to the process of creating a photocollage: “the subject projects different aspects of the self upon a yet-to-be-created puzzle, to then bind them together… creating a form rich in new meanings” (p. 37) [55].

The creative process of collage is characterized by flexibility, as it enables the art maker to explore possible positions and links between visual elements, while crafting them into a new image with new meaning [52]. Digital manipulation also enhanced flexibility [47], since the photos do not need to be glued to the surface, and the composition can always be changed, replaced or even deleted. This flexible, creative and playful approach is essential when exploring the life stories of older adults, because it creates opportunities of retelling and reliving experiences in the continual dynamic process of meaning making [56,57].

In the current study, the creative process of producing an online digital photocollage was oriented by the Dignity Therapy (DT) framework. Dignity Therapy is a brief form of narrative psychotherapy used with clients approaching the end of life [58,59]. It provides a structured process that allows the participants to create and process their life story. The aim of the Dignity Therapy is to increase one’s perceived dignity and meaning in life through the focusing on eight themes: generativity, continuity of self, role preservation, maintenance of pride, hopefulness, aftermath concerns, and care tenor [60]. These themes are explored through a semi-structured interview, in which therapists lead their clients in a process focusing on their most salient moments and roles in life. The interview is then transcribed, and its content negotiated with clients until a final document is produced, the so-called *generative document*. The latter can be gifted to client loved ones as a symbolic legacy and can be supported with images and photographs [61].

Studies have shown how photographs facilitate recollection and allow for a better structuring of the narratives within the Dignity Therapy intervention [24,62,63]. Studies indicate how using photographs as therapeutic tools open up ways to recreate the life stories [64] and engage in the process of meaning making after loss [65,66]. The visual stimuli of the photographs can elicit themes that cannot be expressed by words alone, such as emotions, embodied expressions and muted or sensitive aspects of personal experience [62,63]. Moreover, the photographs, and the connections between them, can be presented in the photocollage as a visual generative document [24].

## 2. Materials and Methods

### 2.1. Research Design

This qualitative study was based on a phenomenological perspective [67] to examine participant subjective experiences during a creative online therapeutic process.

The study design was based on the arts-based approach [68,69] to examine participant experiences through an understanding of their relationship with the artistic expression (in this case, the photocollage) and its creative processes.

### 2.2. Participants

The participants were recruited through social workers at day centers and family members. The inclusion criteria for the study were: (1) 78 years old or over, to include late aging; (2) a Mini-Mental State Examination above 24, to assure normal cognitive performance [70]; (3) the absence of any mental disorder or major depression; (4) visual and hearing abilities to allow participants to engage in an online conversation; (5) access to a computer screen to engage in the creative process (smartphone screens were not appropriate for the creative process of photocollage).

Twenty-four community-dwelling older adults took part in this study, with a mean age of 83.96 (age range: 78–92). There were 12 participants from Italy, with a mean age of 84 (age range: 78–88), and 12 participants from Israel, with a mean age of 83.92 (age range: 80–92). Of this sample, 58.33% were women, 25% lived in couple-hood, all the participants had children, and 58.33% considered themselves religious (for more information see Table 1).

### 2.3. The Creative Process for Online Photocollage

The intervention consisted of three individual 90-min online sessions, in which participants created three separate photocollages. The sessions were conducted through Zoom and operated with a shared screen. The protocol highlighted themes taken from Dignity Therapy. Each session focused on the exploration of one theme: (1) *turning points in the personal narrative*, exploring significant life events that capture the essence and the most important themes, roles, and coping resources in the participant’s narrative [59,71,72]; (2) *personal legacy*, exploring continuity as emerging from the past and being grounded in the values recognized by the individual [24,59]; (3) *future perspective***,** exploring the participant’s wisdom, desires, thoughts and concerns regarding their future and the end of life [73,74,75]. Some meetings were split into two sessions to accommodate the participants. Some of the participants needed to be supported by family members or the adult day center staff in their use of technology such as setting up the computer and the Zoom meetings.

Overall, 78 sessions were conducted with all participants, of which 37 sessions were in Italy, and 42 sessions in Israel. The duration of sessions ranged from 26 to 120 min per session (for the full protocol see Table 2). Occasionally, participants would contact the therapist outside the online setting, via WhatsApp or email, to share thoughts, personal photographs, texts, and ideas they wanted to incorporate into their collage.

At the beginning of each session the participants were presented with a collection of artistic photographs. A different collection was presented in each session. The photographs in this study were taken by Israeli photographers Michal Fattal and Yehudit Liberman and from the University of Haifa collection of photographs at https://www.istockphoto.com/ (last accessed on 3 October 2021). The photographs served as a visual metaphor to stimulate lived experience [76]. The participants were asked to look at the photographs, choose the photographs that stimulated their own personal experiences in relation to the themes raised by the therapists, and arrange them together on a blank space on the screen. This was done with PowerPoint software, which is available on most computers and enables flexibility in terms of the digital creative process of locating, cutting, positioning, and titling the photographs on the blank space. In the current study, the participants were the directors of the creative process, while the therapists physically controlled the activities. Therapists presented the photos, adjusted the size of the photographs, and positioned them on the surface, according to participant’s verbal directions. At the end of the process, the digital photocollages were printed and sent to participants.

Sessions were conducted by the first four authors. The first author is a supervisor and drama therapist specialized in clinical gerontology. The second and third authors were doing their internship in clinical psychology and drama therapy and the fourth author was a master’s degree student in clinical psychology at the time. To ensure adherence to the protocol, the first and fifth authors conducted five training sessions with the team (2.5 h each) prior the intervention. In addition, online 1.5-h supervision sessions were held twice a week during the entire course of the study. All sessions were video-recorded. Participants gave their informed consent before taking part in the intervention and the study was approved both by the Ethics Committee for Experimentation, University of Padua (Confirmation number 0581B1B9C39761AE3C03AD3D93EFDEE9) and the Ethics Committee of the Faculty of Social Welfare and Health Sciences at the University of Haifa, Israel (Confirmation number 366/21). Pseudonyms were used for all participants to preserve confidentiality.

### 2.4. Data Analysis

The video sessions were transcribed and analyzed along with the visual images of the photographs and the photocollages using thematic analysis [77]. The dataset, including the visual images and verbatim transcriptions, was coded by the first four authors, two from Italy and two from Israel. The researchers used Atlas.ti 9 cloud version, which allows for a team of researchers to work and analyze the same data together using a shared code set. All the researchers reviewed and discussed sample quotes during the coding process to ensure that the code definitions were consistent and appropriately applied to the responses. An initial set of codes was created for the dataset, which was followed by an examination of the codes to identify significant broader patterns of meaning, i.e., themes. In the subsequent phase, all candidate themes were checked against the dataset and refined if needed. Next, a detailed analysis of each theme’s content resulted in the definition and final labelling of the themes for the writing up of results.

**Table 2:** Description of the main stages of the intervention.

Stage in the Process	Main Goals	Method and Procedure
Introductory call to participants to introduce the study and its aims	Introductory session between the therapists and participants. Explanation of the process and the technical requirements to conduct the online sessions.	A verbal telephone session.
**Session 1**: Turning Points	Creating a photocollage that visually represents turning points in the life story. These turning points, as the most significant life-events, capture the most important themes and roles in one’s narrative [71]. Exploring one’s most significant life-events as an opportunity to acknowledge the coping resources one has acquired in life, which can also be relevant today, while coping with the COVID-19 pandemic.	Presenting a collection of 60 photographs. The photographs in this study were taken by photographers Michal Fattal and Yehudit Liberman from Israel. Some of the photographs were taken from the University of Haifa’s collection of photographs taken from https://www.istockphoto.com (last accessed on 3 October 2021). Participants were first asked to look at the photographs and see which photographs stimulated personal content, such as life experiences, thoughts, and feelings. Participants were then asked to look at the photographs a second time and choose between 6 to 8 that represented turning points in life or other significant memories. The therapists presented a blank space (a PowerPoint spare slide) and asked the participants how they would like to place the photographs within it. Participants selected one photograph at a time. They placed the photograph in the blank space and in relation to the other photographs of the photocollage. During this process they explained what the photograph represented for them to the therapists and told the stories the photographs elicited. The participants were asked to title each photograph. Once the whole photocollage had been made, the participants were asked to look at the entire photocollage. They were given time for reflection about the things they learned from these turning points in life, and the coping resources they regained. The therapists suggested that they reflect upon the relationship between these coping resources and the ways they could be useful today, while coping with the pandemic. Participants were asked about their experiences during the process of creating their photocollage and their thoughts and feeling about the product.
**Session 2**: Values and Legacy for Future Generations	Creating a photocollage that visually represents the most significant personal values in life that constitute the participants’ legacy for future generations.	Summary of the previous meeting Brief introduction to the task of the second meeting. Presenting a new collection of 60 photographs that was created specifically for this session. Participants were first asked to look at the photographs to see which photographs stimulated personal content, such as life experiences, thoughts, and feelings. Participants were then asked to look at the photographs a second time and choose between 6 to 8 photographs that represented their values, as a legacy they would like to pass down to the younger generations. The therapists presented a blank space (a PowerPoint spare slide) and asked the participants how they would like to position the photographs within the blank space. Participants selected one photograph at a time. They positioned the photograph in the blank space and in relation to the other photographs in the photocollage. During that process they shared what values the photograph represented for them with the therapists and told the stories that were stimulated by the photographs. The participants were asked to title each photograph. Once the whole photocollage had been made, the participants were asked to observe the entire photocollage. They were given time for reflection upon the values that were represented in the photocollage. Participants were presented the 10 values defined in Schwartz’s theory of basic values [78] and some examples of these values. While looking at the photocollage they were asked to identify which Schwartz’s values were present in their photocollage. The therapists suggested reflecting on the relationship between these values and the ways they can be useful today, while coping with the pandemic. Participants were asked about their experiences during the process of creating their photocollage and their thoughts and feeling regarding the product.
**Session 3**: Future Perspectives and Wisdom	Creating a photocollage that visually represents participants’ future perspectives. Reflecting upon the concept of wisdom and how it can be helpful while coping with the COVID-19 pandemic.	Summary of the previous meeting. Brief introduction to the task of the third meeting. Presenting a new collection of 60 photographs that was created specifically for this session. Participants were first asked to observe the photographs to see which photographs stimulated personal content, such as life experiences, thoughts and feelings. Participants were then asked to look at the photographs a second time and choose between 6 to 8 photographs that represented the way they see and wish for their futures and those of their loved ones. The therapists presented a blank space (a PowerPoint spare slide) and asked participants how they would like to position the photographs in the blank space. Participants selected one photograph at a time. They positioned the photograph on the blank space and in relation to the other photographs in the photocollage. During that process they shared what the photograph represented for them and their thoughts and feelings about the future that were stimulated by the photographs. The participants were asked to title each photograph. Once the whole photocollage had been made, the participants were asked to look at the entire photocollage and reflect upon their wishes for their future. Participants shared their thoughts about their own future, including end-of-life issues, and wishes for their loved ones. Participants were asked to reflect about the concept of wisdom and the way it was represented in the photocollage. The therapists suggested reflecting on the relationship between the representations of wisdom and the ways they can be useful today, while coping with the pandemic. Participants were asked about their experience during the process of creating their photocollage and their thoughts and feeling about the product. Participants were asked to observe the three photocollages they created and to give a title to the whole process. Farewell.
Sending a hard copy of the photocollages	Sharing the three photocollages, as a creative generative document for the participants	Printing the three photocollages made by each participant Writing a card thanking them for participating in the study. Sending the three photocollages in an envelope to each participant.

## 3. Results

**Figure 1:**
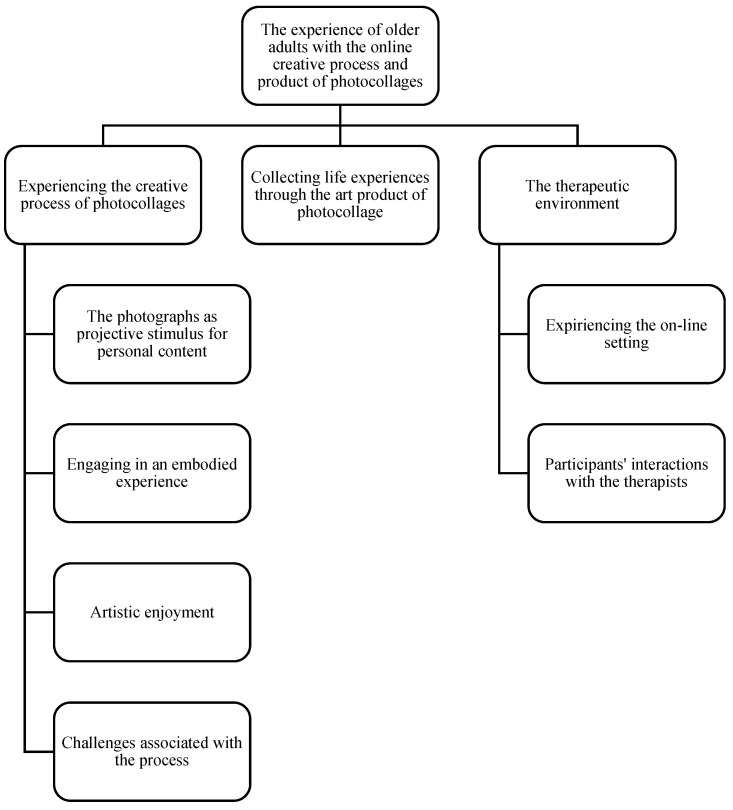
The thematic map.

Three main themes were identified from the experiences reported in the sessions (Figure 1): (1) Experiencing the creative process of photocollages, (2) Collecting life experiences through the art product of photocollage, and (3) The therapeutic environment.

### 3.1. Experiencing the Creative Process of Photocollages: “This Is True Art What We Are Doing Now”

This theme reflected participant experience towards the creative process and the artistic product of the photocollage. Four sub-themes were identified: (1) the photographs as projective stimuli for personal content, (2) engaging in an embodied experience, (3) artistic enjoyment, and (4) challenges associated with the process.

#### 3.1.1. The Photographs as Projective Stimulus for Personal Content

**Figure 2:**
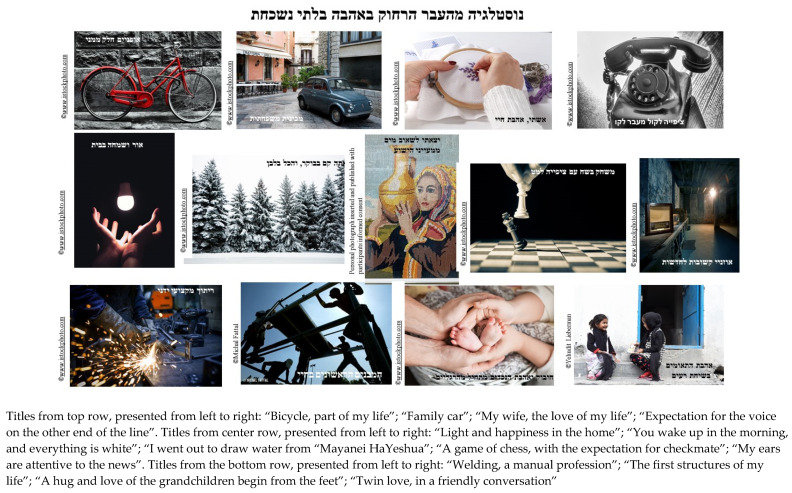
Itzhaks’ third collage titled: “Nostalgia from the far past with unforgettable love”. Reproduced with permission from Michal Fattal, Yehudit Liberman and www.istockphoto.com (accessed on 20 June 2021; 29 June 2021; 3 October 2021).

This sub-theme referred to the projective space provided by the creative process. The participants projected their personal experiences, thoughts and feelings onto the visual images. When asked what he noticed in his final photocollage (see Figure 2), Itzhak, an 85-year-old Israeli man shared his surprise at the memories and stories that were elicited in response to the photographs. His statement shows how the photographs, as a projective stimulus, bypassed conscious experience and evoked memories in a spontaneous way:

I am looking at the (photographs), the bicycle, the telephone, the twins, the building. It gives me, it gives me a sense that I am way in the past. I didn’t even remember these things. All the memories you drew from me, all the memories in my head. You really opened them up. Without (this process) I would have never accessed them … I am surprised. You surprised me. It’s a different aspect of nostalgia. I thought we would focus on (a different part of) my life story. And suddenly, you threw me back to after the army.

**Figure 3:**
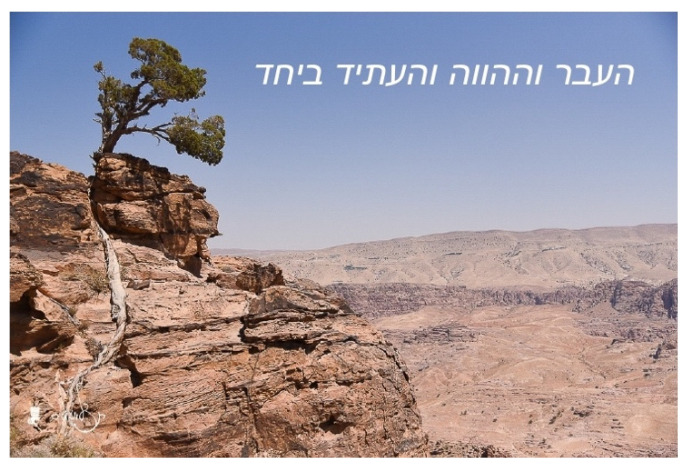
Yossi’s choice of photograph and title: “The past, the present and the future, together”. Reproduced with permission from Yehudit Liberman.

Yossi, an 80-year-old Israeli, chose a photograph of the desert (see Figure 3). His statement shows how the visual image with its colors, shapes and textures served as a metaphor, enabling him to express and process his aging experience and end-of life-issues through symbolic language:

The landscape that I see there, look, look, it’s not only the desert, a crater. There are changes there. Look at the crater, the sand, the earth, the color, the granite. This is all a landscape of how the world changes. This is something. Look at the sand, yellowish-gray in color, the crumbling tree trunk. Thousands of years. This is life, exactly like life. We age, crumble. Crumble and finish. It’s the same, the same. Like a mirror (of life).

**Figure 4:**
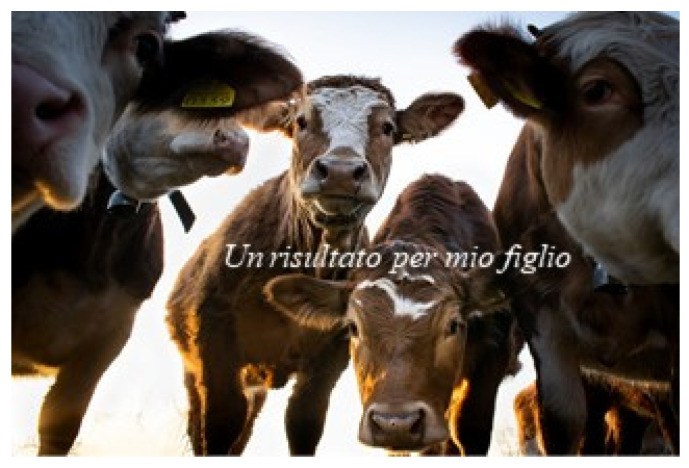
Teresa’s choice of photograph and title: “A product for my son”. Reproduced with permission from www.istockphoto.com (accessed on 29 September 2021).

The visual images of the photographs stimulated memories allowing participants to reminisce about their life experiences. Teresa, a 78-year-old Italian, chose a photograph of a cow (see Figure 4) that triggered a memory that captured parts of her identity. Through this story, she reflected upon the importance of self-direction in her life, as knowledge she wanted to pass on to the therapist:

This one (this photo) for me represents that period in life in which my husband was far away. I told him he should buy me a cow. Because here, in our village, there was a cheese factory, where you could buy milk, and there was this dairy man and so I brought my milk, once a month, and the dairy man made me the cheese. I kept some for my children and the rest I sold so I managed, I was able to provide food for my children.

Mary, a 92-year-old Israeli, shared how the use of visual images as a stimulus created a more positive and protective environment. When asked how the photographs impacted her experience, Mary emphasized the feeling of being safe. This quote illustrates the protective space provided by the aesthetic distancing:

Well, I think the idea of showing pictures in general to evoke whatever the topic is, is a very good idea. I think it leads, for me at least, to an enjoyable experience. With an interview, you sort of have preconceived ideas about what it is you want to know. But with the pictures, it allowed me to be the one to more or less lead where we’re going to go. I didn’t feel invaded. I didn’t feel like sort of attacked in any way. You didn’t invade my privacy. I felt safe.

#### 3.1.2. Engaging in an Embodied Experience

**Figure 5:**
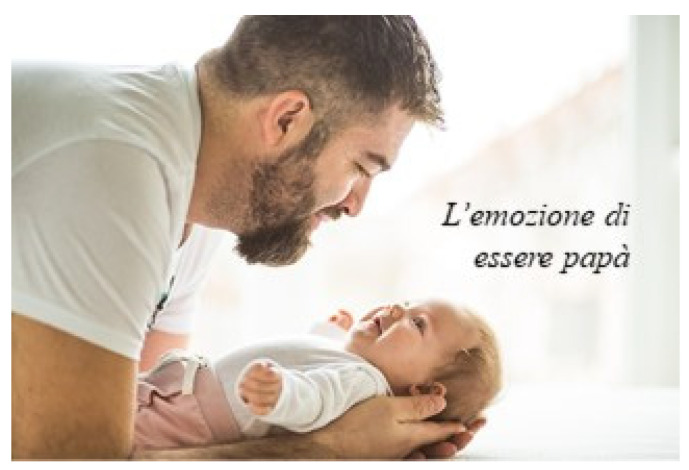
Elena’s choice of photograph and title “The emotion of being a father”. Reproduced with permission from www.istockphoto.com (accessed on 3 June 2021).

This sub-theme referred to the ways the visual images of photographs engaged the participants in an embodied experience. Elena, an 86-year-old Italian woman, said that as opposed to a verbal conversation, the use of visual images stimulated the senses. In this way, an emotional embodied experience could be created. In one collage she referred to a photograph of a man holding a baby and looking into its eyes (see Figure 5). She titled the photograph “The emotion of being a father”, and stated:

Photographs speak. The feeling from this photo, for instance, this dad with his child, you can talk for half an hour but it’s an idea. This is felt, you touch, you touch the emotion with your hand. I find it stimulating, much more than a dialogue, in the sense that photography gives you the emotion. And the emotion, you work with memory, with the reflection. That way, it draws out the true things one has inside.

**Figure 6:**
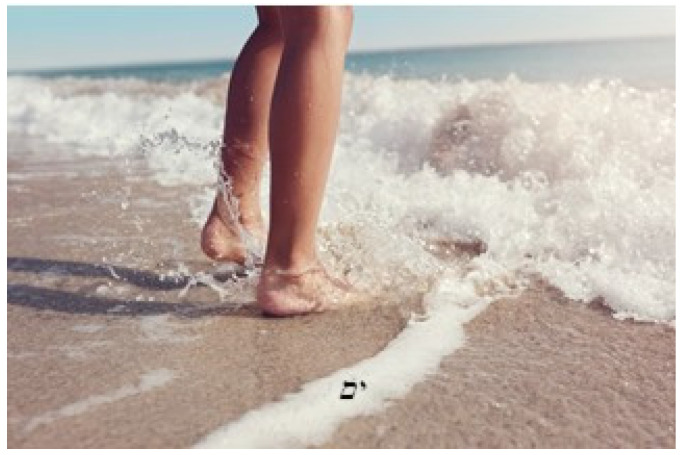
Moshe’s choice of photograph and title “Beach”. Reproduced with permission from www.istockphoto.com (accessed on 29 September 2021).

Another example is presented in the quote of Moshe, an 81-year-old Israeli, who chose a photograph of the ocean (see Figure 6) that triggered a meaningful embodied childhood memory:

Yes, this is part of my life. The first time I went to the beach, I felt the foam in my entire body, not just my feet, I don’t know how to describe it. When the waves break at the shore, it’s like an earthquake on the shore. I felt it, I got emotional. It wasn’t something I had experienced before. The first time I went to the beach I was five maybe six years old.

During the process Mary, a 92-year-old Israeli woman, described how the photographs triggered an embodied experience of emotional inner content: “Here’s well, pictures help trigger a response. A visceral, not visual … visceral from the gut. It’s like the body from inside the body. That’s, that’s a better way of bringing out the feelings.”

The emotional experience was also reflected through embodied responses of participants during the creative process and while observing their final art product of photocollage. When reflecting upon his experience in the third meeting, Itzhak, an 85-year-old Israeli man, shared his embodied reaction:

I am sweating as we are talking about it (future desires). I’m not working hard but just thinking about it makes me sweat. I don’t know if this is natural … it’s a very exciting, emotional topic. It lets me dream, makes me sweat!

#### 3.1.3. Artistic Enjoyment

This sub-theme refers to participant reports during the creative process as being enjoyable and meaningful. Noah, a 91-year-old Israeli, stated that his feelings of enjoyment, while looking at the collection of photographs, derived from both the aesthetics and the content of the photographs that connected him to a deeper level:

It is very beautiful. It reminds me of the way to Jericho, the way to the Dead Sea… The desert, the desert that we see from the amphitheater in Mount Scopus. They (the photos) are all so beautiful, they are all so beautiful. They are all special. They are full of substance, it’s wonderful (laughs).

Similarly, Rivka, an 80-year-old Israeli, addressed the aesthetic quality of the photocollage she created. She felt the movement and connections between photos, which captured her relationship with the photocollage: “There is also aesthetics here, which is also important to me, along with the other values. There is movement, flow. You don’t feel static, you feel movement and flow in the collage, that’s how I feel.”

The creative process also elicited a reflective perspective that made participants think and evaluate their personal experiences. Francesco, an 85-year-old Italian, said: “It was very interesting, and it made my mind think, while doing this small piece of work this morning”. Leonardo, an 85-year-old Italian, described the importance of the creative process and expressed a sense of flow, the feeling that he was fully immersed; he said he did not notice how time had passed: “This (project) is a very important appointment in my life, thank you (name of the researcher). Time flies when I’m with you, it’s marvelous”.

#### 3.1.4. Challenges Associated with the Process

This sub-theme referred to participant challenges while engaging in the creative process. Aaron, an 80-year-old Israeli, stated that the use of photographs in the photocollage was hard for him as they were too abstract. He said it would be easier for him to use words to express his personal story:

Look, I, it’s probably not right for me, this collage. Because I, I like to use words, and, and not something that is so abstract. I can tell you what the, what can be learned from my story. And if you want to, add words to it.

Aaron also mentioned how choosing a limited number of photographs in a short timeframe restricted his ability to create a full and chronological story that reflected his life:

It did not cover life, not a complete life, and not an incomplete one, not even the main chapters. In the pictures there were jumps from, from event to event and from period to period. And there was no order, no logical order. I felt, as I told you, that it was strained. And there were all sorts of holes left in my biography, in my past, that were not covered.

Rivka, an 80-year-old Israeli, stated it was more natural for her to focus on the aesthetic component of the creative process and the artistic product of the photocollage, rather than to connect the visual images to her life story:

It’s harder to link it, philosophically, to points in my life, much harder. What attracts me more is the arrangement, I mean, the perspective, more than connecting life segments to the photographs. I’m making this, the connection, because that’s … we started with that. I mean, you led to it, but it does not come naturally, a little more strained because I understand that there is a purpose to the task of choosing the photographs, it is difficult for me.

Caterina, an 87-year-old Italian, described her struggle with engaging in symbolic conversation. She pointed to a photograph symbolizing the war, but she found it difficult to verbally reflect upon her artistic choice: “This one reminds me of the war, but I don’t know. A specific fallen (soldier). I have no idea … but I would not know how to translate into words”.

### 3.2. Collecting Life Experiences through the Art Product of Photocollage: “Everything Is Here, Life, Everything. Outstanding.”

**Figure 7:**
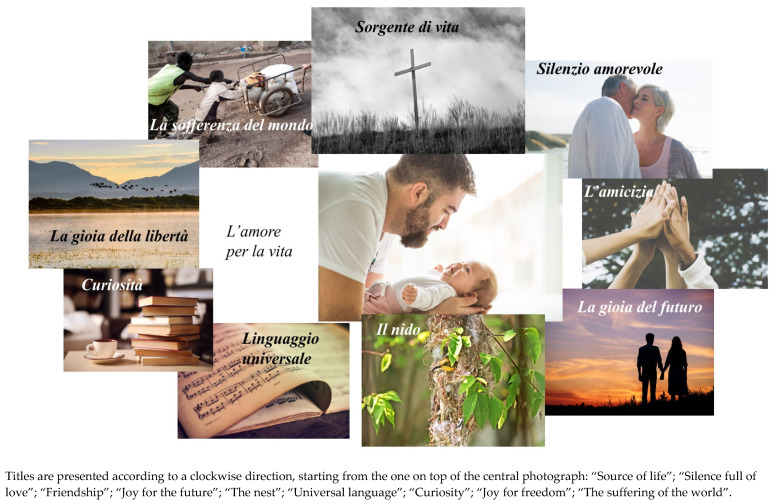
Elena’s photocollage: “Love for life”. Reproduced with permission from www.istockphoto.com (accessed on 5 June 2021; 7 June 2021; 11 July 2021).

This sub-theme reflected how the art process and product of photocollage and the positioning of photos in the collage enabled the process of meaning making. While observing, choosing, and interpreting the photographs, personal experiences, thoughts and feelings were expressed. By positioning the photographs on the surface, participants could link these themes to create meaning. When invited to create a photocollage that represented her values, Elena, an 86-year-old Italian, decided to position a photograph of a man holding a baby in the center of her photocollage (see Figure 7), which she felt corresponded to benevolence and generativity in parenting, as the most meaningful values in her life:

I like this one (this photograph, as the center of my collage) very much, this one of this father holding his child. I like it very much. The importance of a father in the family, especially now when fathers either are too busy working or are unprepared. I think a man who does his job as a father well gives something very valuable to his family. I find this very important.

Elena went on to actively design her photocollage, positioning the photographs in a circular shape around the center photograph and creating a narrative between them:

The cross, let’s put it here or above. Above, above because it’s the most important of all. It covers everything else. That’s it, move the bird. The bird needs to move. That’s it, above the father. It’s as if it embraces everything.

These quotes show how the positioning of the photographs in the collage made it possible for Elena to connect the themes that emerged from the photos. The photocollage presented the most important things in her life in a way that strengthened her positive identity. She titled the collage “Love for life”, as a way to pass her legacy on to the younger generations.

**Figure 8:**
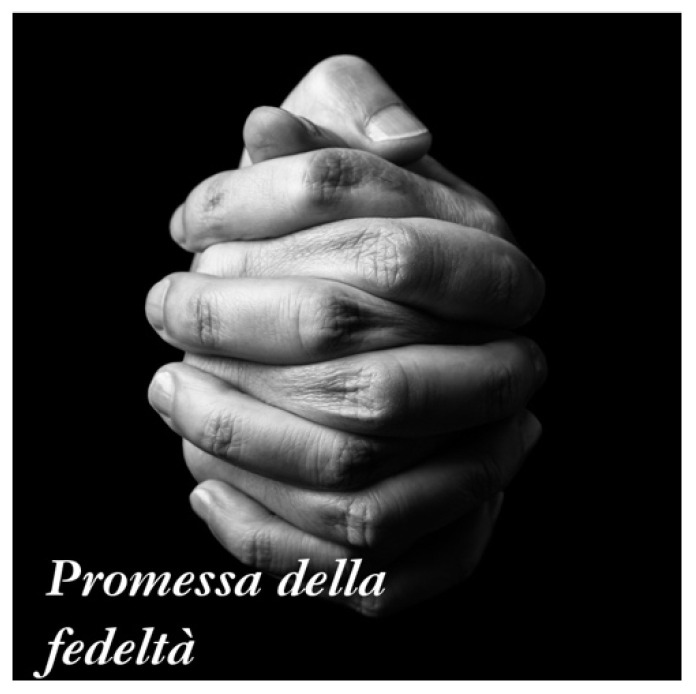
Caterina’s choice of photograph and title: “Promise of loyalty”. Reproduced with permission from www.istockphoto.com (accessed on 29 September 2021).

Frequently, the central photograph in the photocollages represented the most meaningful theme in the life story. It usually represented a central value in life, an accomplishment or a meaningful insight. Many referred to meaningful relationships in life, or the loss of meaningful others. The photo of two hands holding tight (see Figure 8) reminded Caterina, an 87-year-old Italian, of her strong relationship with her husband. The use of the visual images stimulated an embodied representation of their relationship, filling Caterina with a sense of longing for her husband, a strong sense of gratitude for their love and the importance of close relationships in life. She positioned this photograph in the center of her collage and explained:

It’s a promise of loyalty. Because we felt very united, engaged in this thing. You know, also later through the years, when I woke up in the morning, I immediately found my husband’s hand holding mine and then we woke up and did what we had to, but first thing, he held my hand, you know? Therefore, it was very important for us to have this first good morning that we gave each other with our hands, you know? And I am moved when I see these hands holding each other because I spent every morning knowing I had someone who loved me close to me. And I miss him a lot now.

**Figure 9:**
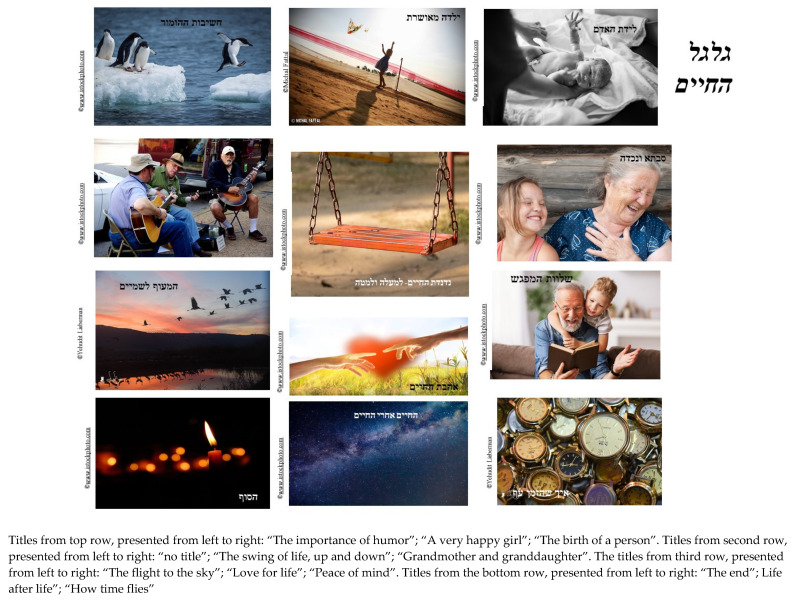
Lea’s photocollage: “The wheel of life”. Reproduced with permission from Michal Fattal, Yehudit Liberman and www.istockphoto.com (accessed on 5 June 2021; 29 June 2021; 16 August 2021; 29 September 2021).

The artwork of Lea, an 83-year-old Israeli, demonstrates the integrative characteristic of photocollage (see Figure 9). She selected and positioned the photos in a way that associated the past, present and future. She started with a photograph of an older woman and a young child. She titled it “Grandmother and granddaughter” and placed it beneath a photograph of a grandfather and explained:

Grandmother and granddaughter. Here I would also put the picture of the grandfather and son, the grandson. Below. A very soothing picture, of a grandfather and grandson talking to each other. I do not know whether to call it the “Generation gap”, or something like that.

Later she added more photographs representing the past and her future and provides some directives on her final moments. She first selected a photograph of clocks that she called “How time flies” and another photograph of birds, which she called “Flight to the sky”—representing her past and the things she had gone through. Then, she positioned a photograph of a burning candle and explained:

The (photograph of the) “flight to the sky”, and the clocks, I would say “how time flies” (...) Yes, this is a good place, at the bottom right side (of the collage). And the candle (at the left side of collage), that is the end. I would like (it) to be in my bedroom, surrounded by my family. And close friends. And they should sing me songs that I like. I could go peacefully.

Lea decided to position the photograph of a swing in the center of the collage, saying: “Up, up and down. The swing of life. You cannot just be up all the time”. When observing the complete photocollage she related to the links between the photographs, and how positioning them side-by-side enhanced her sense of acceptance. She titled the photocollage “The wheel of life” since it contains the past, present and future, the older and younger generations, and the sense of generativity that fills her with meaning. Lea also shared with the therapist that this third collage was her favorite and how discussing the future and seeing everything come together gave her a true sense of peace.

This is the age where I approach the end. My granddaughter on the other hand has just begun her life. I really spend quality time with her. I play with her, tell her stories, read to her, and play. I enjoy her … there is such serenity in this picture. Peace. Okay, you can just (title it) “Peace of mind”.

### 3.3. The Therapeutic Environment: “I Feel like Hugging You”

This theme referred to participant experience in the therapeutic environment. Two sub-themes were identified: (1) the online setting as experienced by the older adults; and (2) participant interaction with the therapists.

#### 3.3.1. Experiencing the Online Setting

This sub-theme referred to the participant experience with the online setting. Some participants stated that the use of the on-line setting and the Zoom application was new to them. Most of them reported a positive experience, and that they got used to the online framework, while others reported a more remote experience. When asked about their experience working with Zoom, Victor, an 81-year-old Italian said it was “very enjoyable. I have always been a little bit, as they say, curious to use all these things (the Zoom)” and Marinella, an 84-year-old Italian complained that “the computer is gibberish to me… (but it is ok if) someone is in front of you. Alone no”, indicating how having the therapist facing her made using the computer easier. Lea, an 83-year-old Israeli said that although she would prefer face to face meetings, she appreciated participating in the study through the Zoom application: “I think the idea, in general, is very nice. And to share through zoom … I don’t see anything bad about it. Yes, to see someone face to face is better, but it wasn’t bad. The way we did it”.

Unlike the previous three participants, Sheila, an 82-year-old Israeli, pointed out some difficulties, while using the Zoom application on the adult day center’s computer:

I’m not used to the computer. I don’t have a computer at home. And it’s as if I’m talking to a wall I don’t know, do you understand? If it was personal, if I met you and we spoke, that’s something else, but to speak to the computer, it makes me feel uncomfortable. When I speak to someone, I see them personally and it makes me feel good.

Some participants reported auditory and vision difficulties during the process which were not necessarily attributable to the online setting. For instance, Costanza, an 84-year-old Italian, said the use of technology in the study did not bother her except for the fact that she had some trouble hearing: “Just because I don’t hear but otherwise … that’s it, it’s (the online setting) just a bit difficult for that reason”.

#### 3.3.2. Participant Interactions with the Therapists

This sub-theme referred to the interaction between participants and therapists during the sessions. Despite the new online experience, the participants described a sense of bonding with the therapist. This was manifested through non-verbal communication, such as laughter and smiles, as well as through the participants explicit comments. Itzhak, an 85-year-old Israeli, described his relationship with his assigned therapist as unique and felt safe enough to express himself as never before: “I’ve never spoken this way. Everything I said in the last hour, I’ve never told anyone like this. I needed it so now it all poured out”. Leonardo, an 85-year-old Italian, expressed his gratitude and positive feelings toward the therapist:

I feel like hugging you, because, this evening, you gave me the joy to express things I had inside. I would wait impatiently for you to call me, I waited, I was impatient… I would like to meet you again; you gave me joy.

Aaron, the 80-year-old Israeli man who described a sense of difficulty with certain aspects of the process, expressed a positive feature of the interaction: “I’m glad that we got along so well. It was nice. Because of you, it will be too bad to end these meetings”.

## 4. Discussion

The present study explored the experiences of older adults who took part in an online therapeutic creative process of creating individual digital photocollages. The intervention was developed based on the CAT theories and methods. It integrated the experiences of both Israeli and Italian older adults. The study was conducted in 2021, when both Israeli and Italian governments were enforcing social distancing measures to reduce the spread of the virus. While for most of the participants this was their first experience in an online setting, and although they were not experienced with online art-based interventions, the findings show that the intervention enabled a creative process via Zoom where the participants were able to process their life experiences in a safe and creative therapeutic environment. Thus, CAT interventions can be made accessible and available to a broader population of older adults, including those who are homebound or have limited access to therapy due to mobility difficulties [39].

The findings suggest that the photographs served as artistic objects that enabled the projection of emotions and thoughts. The visual stimuli supported the participant narratives and engaged them in more symbolic language, which stimulated the expression of the mental content in a more spontaneous way. As in other forms of art therapy, this symbolic language enabled non-verbal aspects of their personal experiences to be expressed [5,6,79]. The process facilitated concretization, since the mental content that was stimulated by the images was transformed into the externalized tangible artistic presentation of the photocollage [7].

The findings also show that the creative process of digital photocollage, incorporating the visual images of photographs, engaged the participants in an embodied sensorial experience, as has been reported in the CAT literature [3,4] and in studies involving the creation of collages [52]. This occurred even though, in the current study, the physical enactment of the art making with its sensory experience was absent in the online setting. This points to the value of the symbolic visual language of the art to engage individuals with their inner emotional embodied experiences, regardless of modality.

For some participants, the distancing created by the projective space generated a safer environment, which echoes the concept of aesthetic distancing in CAT [9]. Specifically, the participants reported they were able to simultaneously be close enough to the experience by affectively identifying with the photographs and the photocollage art product, yet distant enough to cognitively reflect upon it, and achieve new insights. Although some participants reported they had difficulties engaging in a symbolic conversation, or connecting the images to their personal stories, many reported the process to have been a meaningful experience and perceived their final art products as mirroring their life stories. This is consistent with other findings in the CAT literature which show how the existence of an identifiable external artwork enables this distancing of the self, but at the same time enables the individual to recognize the represented self in it [80]. The process also evoked artistic enjoyment. Some participants stated feeling so engaged that they experienced a sense of flow and immersion in the creative process. This positive experience that involves enjoyment and meaningful activity in a social context is essential for the psychological and subjective well-being of the older adults [81].

Crafting the photocollage allowed the participants to process their life stories. The use of the photocollage encouraged the creation of an integrative view of the self, since various life experiences were visualized and integrated within the same art product. The art of collage can produce an intuitive collection of images with inconsistencies and contrasts [52]. Yet, as in Lea’s artwork, assembling visual images and creating associations between them enhanced participant sense of continuity, and generated a more coherent view of self, even in the digital format. In this way, the artistic presentation of photocollage served as an aesthetic container that organized the personal content and imbued it with meaning [82]. Interweaving one’s life story in an integrative coherent manner may thus contribute to strengthening one’s positive identity [54,72].

Hence, the findings show that online arts-based intervention can support one of the common methods in psychotherapy for the aging population, i.e., life story work [83,84], and enrich it with artistic and symbolic language. The creative process also allowed participants to process issues connected with their aging and end-of-life experiences. It followed the themes characterizing psychotherapy in late life [85], indicating the ability of the process to meet the mental needs of the participants. The photocollage gave participants an opportunity to share their wisdom with the younger generations, both narratively, during the session, and through the concrete art product they were given at the end of the intervention, as a generative document to share with their loved ones. The production of a visual generative document can increase participants’ sense of dignity and meaning in life [24], and validate the older adult role in the community.

Finally, most of the participants reported they had a positive experience with the online setting. This is consistent with other studies indicating that older adults can benefit from the on-line psychotherapy [38,39,42] and disconfirms the frequent assumption by psychotherapists that older adults lack the basic skills and familiarity with the online world to be successfully involved in teletherapy interventions [36]. Participant experiences also show that they were able to create a positive and intimate connection with an unfamiliar therapist [86]. Given the pivotal role of social interaction in mental health and in cognitive resilience of older adults [32,87], the findings may indicate that a meaningful interaction and a therapeutic relationship could be achieved in the online setting. The ability to have meaningful and creative sessions that meet the psychological needs of older adults was vital during the COVID-19 social restrictions [88]. This can be generalized to other situations of social isolation, which is considered to be a devastating catalyst for a decline in mental and physical health [41].

## 5. Limitations

The short-term nature of this study, which only lasted three meetings, curtailed the therapeutic process. Some participants indicated that the short time frame restricted their ability to process their life stories fully. A more spontaneous long-term process, which would enable the participants to retrieve their memories, experiences and emotions in their own way, without time limitations or a structured format might be more suitable for some participants. The present study was also limited to older adults with normal cognitive performance, thus excluding individuals with cognitive decline and adaptations for this population. Another limitation is that some participants needed to be supported by family members or staff in their use of technology, such as setting up the Zoom meetings; this might have affected the intervention in ways that were not possible to evaluate.

## 6. Future Research

A longer intervention of the same nature might make it possible to cover the whole life story of older adults more fully. It would allow for a more complete evaluation through the integration of both qualitative and quantitative methods to assess the effects of the intervention on mental health indices. Future research could also evaluate the integration of personal photographs into the collage. An adapted version of this protocol could be conducted with other aging populations such as people in dementia care, for instance, through dyadic interventions involving care-givers [89,90] or in palliative care contexts [91], because it is based on dignity therapy, which is a valuable method for end-of-life interventions [92,93]. In this study, the participants directed the creative process, while the therapists physically controlled the activities. Future studies should also test new technologies and applications that will enable the participants to gain more control over the creative process with more concrete activities such as browsing, cutting, and positioning the photographs on the surface by themselves. Finally, future studies should analyze themes raised by the creative process from an intercultural perspective and explore communalities and differences between Italian and Israelis on issues such as aging, coping resources and end-of-life.

## 7. Conclusions

The present study explored the experiences of older adults who took part in an online therapeutic creative process of digital photocollage during the COVID-19 pandemic. The findings show that the creation of a digital photocollage allowed the participants to reminisce, process their life experiences and create an integrative view of self, which is connected to the acquisition of wisdom in late life. The intervention facilitated a creative process that engaged the participants in an embodied sensorial experience. The process also involved artistic enjoyment along with positive, close interactions with the therapists. The findings suggest that tele-CAT with photocollages can make creative therapeutic processes more accessible to the aging population.

## Figures and Tables

**Table 1:** Participant Demographics.

Variables	Israeli Participants	Italian Participants	Total
Mean age (range)	83.92 (80–92)	84 (78–88)	83.96 (78–92)
Gender	6 females	8 females	58.33% female
Place of birth	3 in Israel; 4 in N. America; 4 in Europe; 1 in Asia	All were from Italy	37.5% had immigrated (only Israeli participants)
Marital status	1 married; 2 divorced; 9 widowed	5 married; 7 widowed	25% married
Education	4 with a high school education; 8 with a college education	6 with a primary school education; 6 with high school education	25% with a primary school education; 41.66% with a high school education; 33.33% with a college education
Religiosity	8 defined themselves as secular; 4 as religious	2 defined themselves secular; 10 religious	58.33% defined themselves as religious
Religion	11 secular Jewish; 1 atheist	All the participants considered themselves Catholic	45.83% defined themselves as secular Jewish; 50% Catholic; 4.16% atheist.

## Data Availability

The data presented in this study are available on request from the corresponding author. The data are not publicly available due to restrictions of privacy.
